# Epidemiology of Buruli Ulcer Infections, Victoria, Australia, 2011–2016

**DOI:** 10.3201/eid2411.171593

**Published:** 2018-11

**Authors:** Michael J. Loftus, Ee Laine Tay, Maria Globan, Caroline J. Lavender, Simon R. Crouch, Paul D.R. Johnson, Janet A.M. Fyfe

**Affiliations:** Victorian Department of Health and Human Services, Melbourne, Victoria, Australia (M.J. Loftus, E.L. Tay, S.R. Crouch);; Austin Health, Heidelberg, Victoria, Australia (M.J. Loftus, P.D.R. Johnson);; Victorian Infectious Diseases Reference Laboratory, Melbourne (M. Globan, C.J. Lavender, J.A.M. Fyfe);; The University of Melbourne, Parkville, Victoria, Australia (P.D.R. Johnson)

**Keywords:** Mycobacterium ulcerans, Buruli ulcer, skin ulcer, tuberculosis and other mycobacteria, epidemiology, Australia

## Abstract

Buruli ulcer (BU) is a destructive soft-tissue infection caused by the environmental pathogen *Mycobacterium ulcerans*. In response to rising BU notifications in the state of Victoria, Australia, we reviewed all cases that occurred during 2011–2016 to precisely map the time and likely place of *M. ulcerans* acquisition. We found that 600 cases of BU had been notified; just over half were in residents and the remainder in visitors to defined BU-endemic areas. During the study period, notifications increased almost 3-fold, from 66 in 2013 to 182 in 2016. We identified 4 BU-endemic areas: Bellarine Peninsula, Mornington Peninsula, Frankston region, and the southeastern Bayside suburbs of Melbourne. We observed a decline in cases on the Bellarine Peninsula but a progressive increase elsewhere. Acquisitions peaked in late summer. The appearance of new BU-endemic areas and the decline in established areas probably correlate with changes in the level of local environmental contamination with *M. ulcerans*.

Buruli ulcer (BU) is a destructive skin and soft tissue infection caused by *Mycobacterium ulcerans*. Although the infection is most prevalent in sub-Saharan Africa, cases have been reported in 33 countries (*1*). The primary risk factor for acquisition of BU is residence in or visitation to a BU-endemic area; however, the environmental reservoirs and modes of transmission within these areas are not completely understood. Recently, researchers have proposed that in Victoria, Australia, possums (arboreal marsupials) are environmental reservoirs and amplifiers (*2*,*3*) and biting insects are mechanical vectors (*4*–*6*).

At least 3 areas in Australia are considered BU endemic: a small far northern region of Queensland near the Daintree Rainforest, the Capricorn Coast of Queensland, and select coastal regions of Victoria (*7*–*10*). We describe the recent epidemiology of all known cases of BU in Victoria that occurred during 2011–2016. We aimed to accurately map current and new BU-endemic areas and compare and contrast the changing incidence in these locations, to document disease severity and associate this with diagnostic delay, and to identify times of increased transmission risk.

## Methods

### Study Population

The study population comprised all case-patients with BU notified to the Victorian Department of Health and Human Services (DHHS) from January 1, 2011, through December 31, 2016. In Victoria, almost all cases of BU are diagnosed or confirmed by PCR performed at 1 reference center (*11*,*12*). A positive PCR is sufficient for notification of a case of BU. All PCR-positive samples were subsequently cultured for reference purposes according to World Health Organization (WHO) guidelines (*13*).

Enhanced surveillance data collection forms have been in use by DHHS since January 1, 2011 (*14*); where required, public health officers obtain additional information through telephone interviews with clinicians or patients. Information obtained includes patient sex, date of birth, residential address, occupation, residence in or travel to BU-endemic areas within the preceding 12 months, date of symptom onset, date of first visit to a healthcare worker, date when BU was first suspected, form(s) of disease (ulcer, papule, nodule, plaque, cellulitis, edema), location of lesion(s), size of affected area including palpable induration (graded by WHO categories I, II, III [*15*]), laboratory results, and treatment details. If enhanced surveillance forms were incomplete or ambiguous with respect to travel to BU-endemic areas, date of symptom onset, or first visit to a healthcare worker, 1 author (M.L.) conducted follow-up telephone interviews. 

### Definitions

We defined a case-patient as a patient with a clinical lesion and a positive *M. ulcerans* PCR or culture result (usually both). We defined BU-endemic areas as suburbs or towns (defined by nationally recognized suburb/locality boundaries [*16*]) where >2 residents had been affected by BU without recalled travel history to another known BU-endemic area in the preceding year; suburbs or towns adjacent to a known endemic area with >1 affected resident or visitor without recalled travel history to any other known endemic areas in the preceding year; or suburbs or towns where *M. ulcerans* had been detected in environmental samples (*2*,*17*). Severe disease was defined as WHO category II or III disease at diagnosis (*14*).

**Figure 1:**
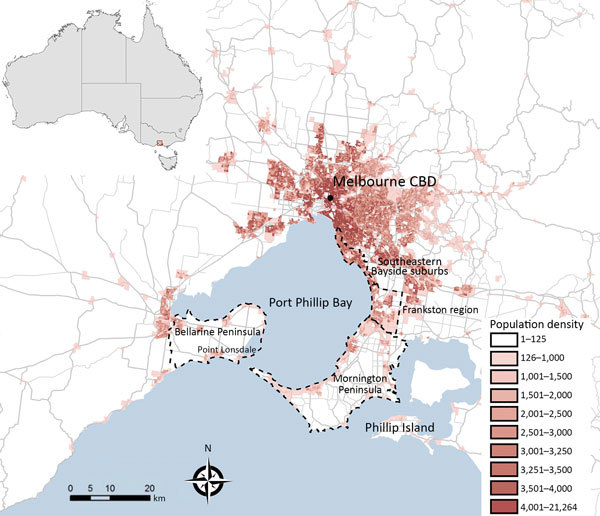
Melbourne, Victoria, Australia, and surrounding areas, showing population density and outlines of the 4 geographic regions used in study of the epidemiology of Buruli ulcer infections in Victoria, Australia, 2011–2016. Population density calculated as residents per square kilometer, according to the Australian Bureau of Statistics 2013 estimated resident population data at the level of Statistical Area Level 1 (*18*). Inset shows location of Melbourne in Australia. CBD, central business district.

BU-endemic areas were further classified by grouping into the following broad geographic regions: Bellarine Peninsula, Mornington Peninsula, Frankston region, and the southeastern Bayside suburbs (Figure 1). A full list of suburbs or towns considered BU endemic and their grouping into categories is available in Technical Appendix Table 1.

We defined residents as case-patients with a postal address in a BU-endemic area or case-patients who reported residing in a BU-endemic area more than half of the year before symptom onset. The remainder were defined as nonresidents. The exception was residents of the Frankston region and southeastern Bayside suburbs; if these case-patients had a history of travel to BU-endemic areas with higher BU incidence rates, their residency status was determined at a case conference as resident, nonresident, or unclear.

We defined likely exposure location (LEL) as the most likely place of BU acquisition, recorded at the suburb and regional levels. In April 2017, four authors (M.J.L., E.L.T., D.D.R.J., J.A.M.F.) held a case conference to allocate residency status and LEL for case-patients for whom these factors were questionable. The panel considered the frequency, duration, and nature of exposures to BU-endemic areas; the timing of exposures relative to symptom onset (considering the average incubation period of 4–5 months [*19**,**20*]); and the relative rates of BU in each location in the year of their exposure. Decisions were reached by consensus.

Confidence in LEL was expressed as definite, probable, or multiple. The term definite was applied to all residents of higher risk BU-endemic areas (Bellarine or Mornington Peninsulas), residents of lower risk BU-endemic areas (Frankston region or the southeastern Bayside suburbs) without significant travel history to other BU-endemic areas, and nonresidents who had traveled to only 1 BU-endemic area. The term probable was applied to residents of the Frankston region or the southeastern Bayside suburbs or nonresidents with exposure to >2 BU-endemic areas where 1 location was clearly most likely to be responsible. The term multiple was applied to residents of the Frankston region or southeastern Bayside suburbs and to nonresidents with exposure to >2 BU-endemic areas where no location was considered more likely than another to be responsible.

Some cases could be assigned different degrees of confidence between the suburb and region of exposure. For instance, someone who traveled to many towns within the Mornington Peninsula could be classified as having had multiple exposures at the suburb level but definite exposure at the regional level. If a patient had also been exposed to interstate or overseas BU-endemic regions, where possible, we performed variable-number tandem-repeat typing on the isolate to identify the region of origin (*21*).

### Statistical Analyses

We performed descriptive analyses of data and reported means or medians, depending on their distribution. Rates were calculated by using the Australian Bureau of Statistics midyear estimated resident population data (*22*). To explore significant associations between groups, we used χ^2^ or Fisher exact tests for categorical variables and Mann-Whitney U or Kruskal-Wallis tests to compare the time between symptom onset and first healthcare visit and between symptom onset and BU diagnosis between groups. We used the Edwards test for seasonal trends (*23*) to determine the periodicity of monthly totals of date of symptom onset, first visit to a healthcare worker, and diagnosis. We investigated the predictors of disease severity by using logistic regression. Independent variables with p<0.2 in univariate analysis were considered in multivariate analysis.

Maps were prepared by using an ESRI ArcGIS server version 10.4.1, accessed from Geocortex Essential online mapping software (http://www.geocortex.com). All data preparation and analyses were conducted by using Microsoft Excel 2010 (Redmond, WA, USA) and Stata version 13.0 (StataCorp LLC, College Station, TX, USA).

### Ethics Statement

DHHS officers obtained all identifying data in this study under the legislative authority of the Public Health and Wellbeing Act 2008, Victoria. This act covers notifiable diseases; separate ethics approval was not required.

## Results

### Demographics and Rates of Disease

**Figure 2:**
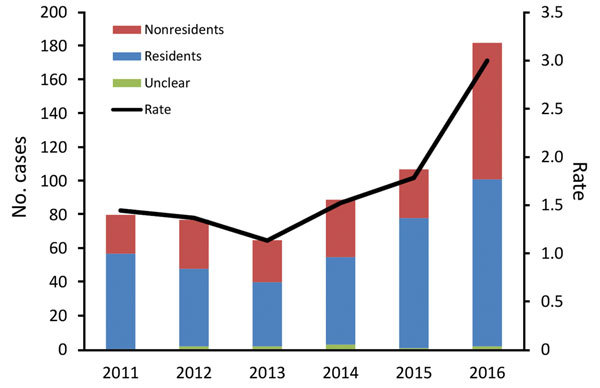
Number of Buruli ulcer cases and incidence rate (no. cases/100,000 persons), by year and resident status, Victoria, Australia, 2011–2016.

During 2011–2016, a total of 600 BU cases were notified to DHHS; annual case numbers increased from 66 cases in 2013 to 182 cases in 2016 (Figure 2). During March and April 2017, telephone interviews were required to obtain further information for 117 (19.5%) case-patients regarding their travel history and LELs; these calls were completed for 98 (83.8%) of 117 contactable case-patients.

Of the 600 case-patients, 342 (57.0%) were male; the median age was 54 years (range 1–95 years). In terms of residency, 16 case-patients were considered residents of the Frankston region or southeastern Bayside suburbs but had substantial exposures to other higher risk BU-endemic areas; 6 were considered nonresidents, and residency status for 10 was deemed unclear. Thus, the 10 case-patients with unclear status were excluded from subsequent analyses of residents versus nonresidents. Of the remaining 590 case-patients, 369 (61.5%) were classified as residents and 221 (36.8%) as nonresidents of BU-endemic areas. On average, residents were much older (median age 58 years) than nonresidents (median age 44 years) (p<0.0001) and accounted for over half of all cases each year, varying between a low of 54.4% in 2016 and a high of 72.0% in 2015 (Figure 2).

The overall rates in Victoria were 1.1–3.0 cases/100,000 population (Figure 2). However, because of the focal distribution of BU, rates for certain suburbs or towns were significantly higher. For instance, for residents of the adjoining towns Rye and Tootgarook on the Mornington Peninsula, the combined rate was 366 cases/100,000 population in 2016, which is >100-fold higher than the state average. Across the 6-year study period, the rates of BU among residents of the 4 geographic regions were 7.6 cases/100,000 population on the Bellarine Peninsula, 3.1 cases/100,000 population on the Mornington Peninsula, 1.1 cases/100,000 population in the Frankston region, and 0.6 cases/100,000 population in the southeastern Bayside suburbs.

### LELs at the Regional Level

Of the 600 case-patients, 565 (94.2%) had LELs at the regional level defined as definite, 13 (2.2%) as probable, and 18 (3.0%) as multiple. For 3 case-patients—all residents of metropolitan Melbourne—no exposure to a BU-endemic area was reported, and for 1 case-patient, no information regarding travel was available.

**Figure 3:**
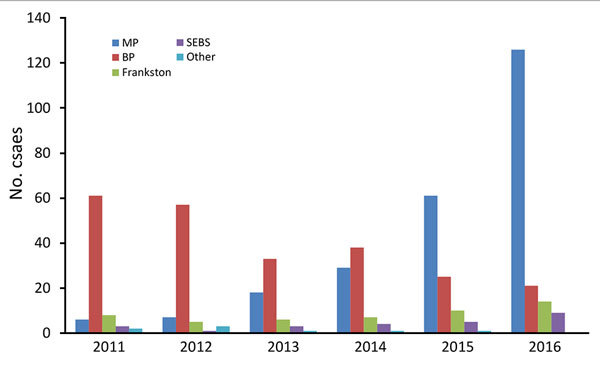
*Mycobacterium ulcerans* likely exposure locations, by region and year (definite cases only), Victoria, Australia, 2011–2016. BP, Bellarine Peninsula; MP, Mornington Peninsula; SEBS, southeastern Bayside suburbs.

**Figure 4:**
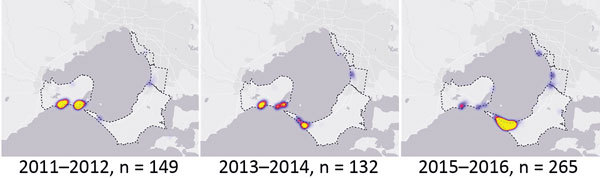
Heat maps of *Mycobacterium ulcerans* likely exposure locations (definite cases only), by 2-year period, Victoria, Australia, 2011–2016.

The most common LELs by region were the Mornington Peninsula (247/565, 43.7% of definite exposures) and the Bellarine Peninsula (235, 41.6%), followed by the Frankston region (50, 8.8%) and the southeastern Bayside suburbs (25, 4.4%) (Figure 3). Although the Mornington and Bellarine Peninsulas were linked to a similar number of LELs over the entire study period, their patterns differed greatly (Figure 4). LELs were highest for the Bellarine Peninsula in 2011 (61 LELs) and steadily declined to only 21 in 2016. In contrast, LELs were initially very few for the Mornington Peninsula (only 31 in the first half of the study period) and increased substantially from 2015 on. More than half of all LELs linked to the Mornington Peninsula were notified in 2016 alone.

When the 31 case-patients with probable or multiple exposures were also considered, LELs linked to the southeastern Bayside suburbs and Frankston region increased disproportionately (Bayside suburbs, 48% increase, from 25 to 37 exposures; Frankston, 22% increase, from 50 to 61). Increases for the Mornington Peninsula (11%, from 247 to 273) and the Bellarine Peninsula (6%, from 235 to 249) were smaller (Technical Appendix Table 2).

### Date of Symptom Onset

**Figure 5:**
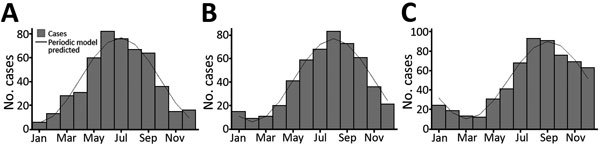
Timing of A) symptom onset, B) first visit to a healthcare worker, and C) diagnosis of Buruli ulcer for patients with *Mycobacterium ulcerans* infection, Victoria, Australia, 2011–2016.

Information regarding the date of symptom onset was available for 494 (82.3%) of the 600 patients and regarding the date of first visit to a healthcare worker for 497 (82.8%) patients. With regard to date of symptom onset, the monthly variation was best described by a simple harmonic curve (p<0.0001), which showed a trough in early January (summer in the Southern Hemisphere) and a peak in early July (winter in the Southern Hemisphere). Onset of symptoms was lowest in January (summer, 6 cases) and highest in June (winter, 82 cases) (Figure 5, panel A). The same seasonal pattern was found for residents and nonresidents; peaks and troughs occurred in identical months.

The monthly variation in the dates of first visit to a healthcare worker and diagnosis of BU were also best described by simple harmonic curves (p<0.0001 for both). Peaks for visit to a healthcare worker occurred in mid-August and for BU diagnosis in mid-September (Figure 5, panels B, C).

### Clinical Features

The site of BU lesion was available for 585 (97.5%) of the 600 case-patients; among these, the most common site of infection was the lower limbs (424/585, 72.4%). More than two thirds of those lesions (51.0% overall) were below the knee; the remainder were predominantly on the upper limbs (23.3%), and a small proportion were on the torso (0.7%) or head and neck (0.8%) (Tables 1, 2). Univariate analysis revealed no significant associations between site of infection and patient age or sex. Lesion location has recently been accurately mapped in a separate study of patients with BU in Victoria (*24*).

**Table 1:** Clinical site and severity, delays to first healthcare visit and diagnosis, and management strategies for Buruli ulcer case-patients, by age and region of exposure, Victoria, Australia, 2011–2016*

Variable	Total no. (%)	Age group, y					Exposure, definite at area level, n = 565			
<15	15–60	>60	p value†	BP	MP	Other	p value†
Total	600	72 (12.0)	285 (47.5)	243 (40.5)			235 (39.2)	247 (41.2)	83 (13.8)	
Lesion site, total no.	585	71	278	236	0.381‡		228	240	82	0.003‡
Lower limb	424 (72.5)	52 (70.3)	210 (75.5)	162 (68.6)			154 (67.5)	182 (75.8)	58 (70.7)	
Below knee	306 (52.3)	31 (41.9)	149 (53.6)	126 (53.4)			109 (47.8)	129 (53.8)	45 (54.9)	
Knee or above	86 (14.7)	16 (21.6)	48 (17.3)	22 (9.3)			33 (14.5)	39 (16.3)	10 (12.2)	
Unspecified	32 (5.5)	5 (6.8)	16 (5.8)	14 (5.9)			12 (5.3)	14 (5.8)	3 (3.7)	
Upper limb	140 (23.9)	17 (23)	61 (21.9)	62 (26.3)			71 (31.1)	45 (18.8)	19 (23.2)	
Other sites	12 (2.1)	1 (1.4)	6 (2.2)	5 (2.1)			2 (0.9)	9 (3.8)	1 (1.2)	
Head/neck	5 (0.9)	1 (1.4)	2 (0.7)	2 (0.8)			1 (0.4)	4 (1.7)	0 (0.0)	
Torso	4 (0.7)	0 (0)	2 (0.7)	2 (0.8)			1 (0.4)	2 (0.8)	1 (1.2)	
Buttock/perineum	3 (0.5)	0 (0)	2 (0.7)	1 (0.4)			0	3 (1.3)	0	
>1 site	9 (1.5)	1 (1.4)	1 (0.4)	7 (3.0)			1 (0.4)	4 (1.7)	4 (4.9)	
WHO category, total no.	523	64	244	215	0.041		198	221	69	0.219
I	415 (79.3)	53 (82.8)	204 (83.6)	158 (73.5)			166 (83.8)	171 (77.4)	52 (75.4)	
II	71 (13.6)	7 (10.9)	30 (12.3)	34 (15.8)			23 (11.6)	34 (15.4)	9 (13.0)	
III	37 (7.1)	4 (6.3)	10 (4.1)	23 (10.7)			9 (4.5)	16 (7.2)	8 (11.6)	
Time to healthcare visit					0.0146					0.2770
Median (IQR), d	28 (14–60)	21 (14–36)	30 (14–60)	21 (14–49)			28 (14–42)	30 (14–60)	30 (14–60)	
Time to diagnosis					0.1536					0.0001
Median (IQR), d	63 (35–109)	63 (39–122)	65 (37–114)	54 (31–103)			46 (27–83)	70 (37–119)	82 (21–127)	
Treatment, total no.	517	65	227	214	0.606		205	210	69	0.281
Antimicrobial drugs only	328 (63.4)	41 (63.1)	145 (63.9)	142 (66.4)			137 (66.8)	135 (64.3)	37 (53.6)	
Surgery only	27 (5.2)	2 (3.1)	16 (7)	9 (4.2)			12 (5.9)	9 (4.3)	5 (7.2)	
Antimicrobial drugs and surgery	162 (31.3)	22 (33.8)	77 (33.9)	63 (29.4)			56 (27.3)	66 (31.4)	27 (39.1)	
Surgery, total no.	165	19	81	65	0.621		59	66	26	0.671
Debridement	54 (32.7)	6 (31.6)	29 (35.8)	19 (29.2)			20 (33.9)	23 (34.8)	5 (19.2)	
Narrow excision	53 (32.1)	8 (42.1)	26 (32.1)	19 (29.2)			19 (32.2)	20 (30.3)	10 (38.5)	
Wide excision	58 (35.2)	5 (26.3)	26 (32.1)	27 (41.5)			20 (33.9)	23 (34.8)	11 (42.3)	

*Values are no. (%) patients except as indicated. BP, Bellarine Peninsula; IQR, interquartile range; MP, Mornington Peninsula; WHO, World Health Organization. †p value provides comparisons across all categories. ‡p value provides comparison between lower limb, upper limb, all other sites, and patient age or exposure location.

**Table 2:** Clinical site and severity, delays to first healthcare visit and diagnosis, and management strategies for Buruli ulcer case-patients, by sex, Victoria, Australia, 2011–2016*

Variable	Male	Female	p value†
Total no.	342 (57.0)	258 (43.0)	
Lesion site, total no.	333	252	0.116‡
Lower limb	231 (69.4)	193 (76.6)	
Below knee	167 (50.2)	139 (55.2)	
Knee or above	39 (11.7)	47 (18.7)	
Unspecified	25 (7.5)	7 (2.8)	
Upper limb	87 (26.1)	53 (21)	
Other sites	8 (2.4)	4 (1.6)	
Head/neck	2 (0.6)	3 (1.2)	
Torso	4 (1.2)	0 (0.0)	
Buttock/perineum	2 (0.6)	1 (0.4)	
>1 site	8 (2.4)	2 (0.8)	
WHO category, total no.	295	228	0.078
I	226 (76.6)	189 (82.9)	
II	42 (14.2)	29 (12.7)	
III	27 (9.2)	10 (4.4)	
Time to healthcare visit			0.6153
Median (IQR), d	29 (14–60)	28(14–42)	
Time to diagnosis			0.5134
Median (IQR), d	63 (35–109)	65(36–117)	
All treatment, total no.	296	221	0.03
Antimicrobial drugs only	189 (63.9)	139 (62.9)	
Surgery only	9 (3.0)	18 (8.1)	
Antimicrobial drugs and surgery	98 (33.1)	64 (29)	
Surgical treatment, total no.	91	74	0.331
Debridement	33 (36.3)	21 (28.4)	
Narrow excision	25 (27.5)	28 (37.8)	
Wide excision	33 (36.3)	25 (33.8)	

*Values are no. (%) patients except as indicated. IQR, interquartile range; WHO, World Health Organization. †p value provides comparisons across all categories. ‡p value provides comparison between lower limb, upper limb, all other sites, and patient sex.

Information regarding the form of disease was available for 536 (89.3%) case-patients. Only 1 form of disease was documented for most patients, 2 forms for 12 patients, and 3 forms for 1 patient. The most common form of disease was an ulcer (455/536, 84.9%), followed by a papule (37, 6.9%), a nodule (22, 4.1%), cellulitis (20, 3.7%), plaque (8, 1.5%), or edema (7, 1.3%).

Overall, 415 (69%) patients had WHO category I disease, 71 (11.8%) category II, and 37 (6.2%) category III. For the remaining 77 (12.8%), no information regarding disease severity was available (Tables 1, 2). No relationship was found between severe disease and year of BU diagnosis (Technical Appendix Table 3). Multivariable analysis revealed an increased likelihood of severe BU disease among older patients (>60 years) (odds ratio [OR] 2.19, 95% CI 1.38–3.47) and visitors to BU-endemic regions (OR 1.87, 95% CI 1.16–3.02) (Table 3). Although exposure to BU-endemic regions other than the Bellarine and Mornington Peninsulas seemed to be associated with an increased risk for severe disease, this association did not reach statistical significance (OR 1.997, 95% CI 0.98–3.94). No association with patient sex was found.

**Table 3:** Logistic regression model showing adjusted and unadjusted associations between identified factors and severe Buruli ulcer disease, Victoria, Australia, 2011–2016*

Factor	Disease severity, no. (%) patients†			Univariate model			Multivariate model	
Category I	Categories II and III	OR (95% CI)	p value	aOR (95% CI)	p value
Age, y								
<60	257 (83.4)	51 (16.6)		1.00				
>60	158 (73.5)	57 (26.5)		1.82 (1.19–2.79)	0.006		2.19 (1.38–3.47)	0.001
Sex								
F	189 (82.9)	39 (17.1)		1.00				
M	226 (76.6)	69 (23.4)		1.48 (0.96–2.29)	0.079		1.36 (0.85–2.16)	0.199
Residence								
Resident	251 (82.3)	54 (17.7)		1.00				
Visitor	156 (75.0)	52 (25.0)		1.55 (1.01–2.38)	0.046		1.87 (1.16–3.02)	0.01
Unclear	8 (80.0)	2 (20.0)		1.16 (0.24–5.63)	0.852		NC	
Exposure								
Bellarine Peninsula	166 (83.8)	32 (16.2)		1.00				
Mornington Peninsula	171 (77.4)	50 (22.6)		1.52 (0.93–2.48)	0.097		1.35 (0.81–2.24)	0.244
Other	52 (75.4)	17 (24.6)		1.70 (0.87–3.30)	0.120		2.00 (0.87–3.94)	0.056
Time to diagnosis, d‡				1.002 (0.999–1.005)	0.254			

*aOR, adjusted odds ratio; NC, not calculable because there were no data; OR, odds ratio.  †Severe disease is defined as World Health Organization category II or III. ‡Not included in the logistic regression model because it did not meet the prespecified threshold for significance of p<0.2.

### Time to Diagnosis

All 600 cases of BU were confirmed with PCR testing. Culture results were available for 489 patients; results for 419 (85.7%) were positive for *M. ulcerans*. For some case-patients, the diagnosis of BU was first considered only after being indicated by histologic examination of surgical specimens and later confirmed by PCR.

The median time between symptom onset and first visit to a healthcare worker was 28 days. This time did not differ significantly between residents and nonresidents (p = 0.5253), and although delay to first healthcare visit was shorter for Bellarine Peninsula residents (21 days) than for Mornington Peninsula residents or all other residents, this difference was not significant (p = 0.185 and p = 0.174, respectively) (Table 4). Case-patients 15–60 years of age sought healthcare significantly later (median 30 days) than did younger (<15 years) (21 days, p = 0.021) or older (>60 years) (21 days, p = 0.0179) case-patients (Table 1).

**Table 4:** Demographic and clinical data and management strategies for Buruli ulcer case-patients, by resident status, Victoria, Australia, 2011–2016*

Characteristic	Residents					Nonresidents		Overall
All	BP	MP	p value†	All	p value‡
Total no. cases	369	146	151			221		600
Age, y								
Median (IQR)	58 (38–71)	55 (33–71)	59 (40–71)	0.7081		44 (24–65)	<0.0001	54 (31–9)
<15	32 (8.7)	11 (7.5)	13 (8.6			39 (17.6)		72 (12)
15–60	168 (45.5)	72 (49.3)	65 (43)			110 (49.8)		285 (47.5)
>6	169 (45.8)	63 (43.2)	73 (48.3)	0.555		72 (32.6)	<0.0001	243 (40.5)
Sex								
M	213 (57.7)	79 (54.1)	94 (62.3)			124 (56.1)		258 (43)
F	156 (42.3)	67 (45.9)	57 (37.7)	0.155		97 (43.9)	0.701	342 (57)
Year of diagnosis, total no.	369	146	151			221		600
2011	57 (15.4)	43 (29.5)	4 (2.6)			23 (10.4)		80 (13.3)
2012	46 (12.5)	34 (23.3)	4 (2.6)			29 (13.1)		77 (12.8)
2013	38 (10.3)	16 (11)	13 (8.6)			25 (11.3)		65 (10.8)
2014	52 (14.1)	24 (16.4)	16 (10.6)			34 (15.4)		89 (14.8)
2015	77 (20.9)	18 (12.3)	46 (30.5)			29 (13.1)		107 (17.8)
2016	99 (26.8)	11 (7.5)	68 (45)	<0.0001		81 (36.7)		182 (30.3)
WHO category, total no.	305	118	127			208		523
I	251 (82.3)	102 (86.4)	103 (81.1)			156 (70.6)		415 (79.3)
II	35 (11.5)	13 (11)	15 (11.8)			35 (15.8)		71 (13.6)
III	19 (6.2)	3 (2.5)	9 (7.1)	0.338		17 (7.7)	0.129	37 (7.1)
								
Median time to seeking healthcare, d (IQR)	28 (14–60)	21 (14–42)	28 (14–60)	0.1853		30 (14–60)	0.5253	28 (14–60)
Median time to diagnosis, d (IQR)	48 (27–94)	34 (22–62)	51 (29–95)	0.0016		79 (48–123)	<0.0001	63 (35–109)
Treatment, total no.	301	124	129			206		517
Antimicrobial drugs only	208 (69.1)	90 (72.6)	86 (66.7)			115 (55.8)		328 (63.4)
Surgery only	17 (5.6)	7 (5.6)	5 (3.9)			10 (4.9)		27 (5.2)
Antimicrobial drugs and surgery	76 (25.2)	27 (21.8)	28 (21.7)	0.844		81 (39.3)	0.003	162 (31.3)
Surgical treatment, total no.	78	27	27			83		165
Debridement	27 (34.6)	13 (48.1)	10 (37)			26 (31.3)		54 (32.7)
Narrow excision	26 (33.3)	6 (22.2)	10 (37)			24 (28.9)		53 (32.1)
Wide excision	25 (32.1)	8 (29.6)	7 (25.9)	0.482		33 (39.8)	0.592	58 (35.2)

*Values are no. (%) patients except as indicated. BP, Bellarine Peninsula; IQR, interquartilie range; MP, Mornington Peninsula; WHO, World Health Organization. †p value provides comparison between Bellarine Peninsula residents and Mornington Peninsula residents. ‡p value provides comparison between all residents and nonresidents.

The overall median delay from symptom onset to BU diagnosis was 63 days. Diagnoses were made far sooner for residents (median delay 48 days) than for nonresidents (median delay 79 days) (p<0.0001). The median delay to diagnosis was even shorter for Bellarine Peninsula residents, only 34 days (p<0.0001) (Table 4). Regardless of resident status, the average diagnostic delay for case-patients with definite LELs on the Bellarine Peninsula was shorter (46 days) than for those with definite LELs on the Mornington Peninsula (70 days) or other areas (82 days) (p = 0.0001). The longer delay to diagnosis among nonresidents, and those not exposed on the Bellarine Peninsula, was primarily driven by the delay between first visit to a healthcare worker and receiving the diagnosis of BU (Tables 1, 3).

### Treatment

Treatment information was available for 517 (86.1%) patients. Of these, 490 (94.8%) received antimicrobial drugs; however, specific details of chosen agents and duration of regimen were inconsistently reported on enhanced surveillance forms and therefore were not analyzed. Surgery was performed for 189 (36.6%) of these 517 case-patients; 162 (85.7%) concomitantly received antimicrobial drugs, and 27 (14.3%) underwent surgery only. Women were more likely than men to undergo surgery only (p = 0.01) (Tables 1, 3).

## Discussion

BU is a geographically restricted infection that occurs as local outbreaks. The environmental reservoir and mode of transmission to humans who enter BU-endemic regions is of intense scientific interest because primary prevention will not be possible until these issues have been definitively resolved.

In our epidemiologic investigation, we established that BU incidence is increasing in the temperate state of Victoria, Australia, but that this increase is not uniform. Previously, the most BU-endemic region in Victoria has been the Bellarine Peninsula (*4*,*8*), where incidence is now progressively falling but has been matched by the appearance of new BU-endemic foci on the Mornington Peninsula and in the southeastern Bayside suburbs of Melbourne. Rather than describing a single epidemic of BU in Victoria, it is probably more accurate to propose that we are observing 3–4 distinct outbreaks with different dynamics.

We found a clear seasonal pattern in the timing of symptom onset —a peak in midwinter and a trough in midsummer—consistent with trends shown in previous reports from Victoria (*8*,*25**,**26*). Given the median incubation period for BU in Victoria of 4.5 months (*19**,**20*), most infections there are probably acquired in summer and autumn. Among nonresidents, this trend could be explained purely by increased tourism to beachside BU-endemic regions during warmer months. However, it is noteworthy that seasonality in symptom onset among residents, who were exposed to BU-endemic areas throughout the year, was identical. This finding suggests that transmission risk is specific to the warmer months, perhaps relating to a vector (e.g., higher mosquito numbers), seasonal rainfall patterns or warmer temperatures, or human behavior (e.g., wearing less clothing, spending more time outside) (*24*).

Delays to diagnosis were significantly shorter among residents of BU-endemic areas than among nonresidents and among Bellarine Peninsula residents than among Mornington Peninsula residents. This finding is broadly consistent with previously reported delays of 28 days among Bellarine Peninsula residents and 72 days among nonresidents (*25*) or 42 days among a cohort consisting predominantly of Bellarine Peninsula residents (*8*). Diagnostic delays may be driven by delays in seeking healthcare, delays in healthcare professionals making the correct diagnosis, or both. In our cohort, large discrepancies in overall diagnostic delay between patient groups seemed to be driven primarily by delays in making the correct diagnosis after patients visited a doctor. Our findings probably reflect patients and general practitioners in non–BU-endemic or recently BU-endemic areas being relatively unfamiliar with BU compared with those in more established BU-endemic areas and subsequently delaying the initiation of BU-specific diagnostic testing (such as PCR). Several public health campaigns have been introduced to increase awareness of BU on the Bellarine Peninsula, and further research is under way to clarify other specific factors associated with diagnostic delays in Victoria (*27*).

When calculated across the entire cohort, diagnostic delay was not associated with more severe disease. This finding is somewhat counterintuitive because in the absence of appropriate therapy, most lesions will enlarge and progress over time. However, this cohortwide analysis may be confounded by unaccounted differences among case-patients in the aggressiveness of their disease, their healthcare-seeking behavior, and our inability to control for other factors such as concurrent conditions or immunosuppression. For instance, someone with a rapidly progressive lesion may seek healthcare sooner, be tested more proactively, and receive an early diagnosis of severe disease, whereas another patient with a slowly progressive lesion may seek healthcare later, undergo fewer investigations, and receive a diagnosis after many months with milder disease. Although we found no signal at the population level that diagnostic delay was associated with worse outcomes, public health awareness programs to encourage patients and local doctors to consider BU early remain reasonable.

A recent article, published after our manuscript was submitted, describes clinical information for 426 BU patients at a single tertiary center on the Bellarine Peninsula from 1998 through May 2017 (*28*). Of note, those authors report increasing severity of disease over time, a finding that was not replicated in our more epidemiologically focused analysis, albeit over a much shorter time (Technical Appendix Table 3). Although cases overlap between these 2 articles, our analyses included all notified cases occurring in a 6-year period, which reduces potential referral bias. However, our clinical information was obtained via telephone interview, whereas Tai et al. directly assessed their patients, providing them with more detailed clinical information than we had access to (*28*).

We have previously shown a close association between BU in humans and outbreaks of BU in possums on the Bellarine and Mornington Peninsulas (*2*,*17*). Possums are arboreal marsupials that are common in Victoria, in BU-endemic and nonendemic areas. On the basis of our previous work, we think the changing epidemiology of BU reported here is driven primarily by new outbreaks of BU in local possum populations with spillover to humans mediated by environmentally contaminated biting insects (*4*,*24*,*29*). Of note, the window of human exposure may be quite short; a recent study reported 10 family clusters in which transmission to family members occurred almost simultaneously (*30*).

Limitations of our study include the use of retrospective surveillance data, the long incubation period of BU that introduces recall bias, the contiguous nature of BU-endemic regions, and the shifting geographic distribution of BU, all of which made it challenging to allocate LELs precisely. Although we can use census data for rates of diseases in local populations, the unquantified high levels of tourism to BU-endemic regions during summer make it impossible to accurately estimate the true population at risk or to give a quantitative estimate of risk per day of exposure for visitors.

Although BU is rarely life-threatening, the extent of illness (*31*) and economic costs (*32*) are substantial and increase among patients with larger and more advanced lesions. The findings of our study can guide public health responses. The shorter diagnostic delay among residents of the Bellarine Peninsula suggests that increased community awareness leads to earlier disease detection. Furthermore, understanding the seasonality of BU can guide the optimum timing of different public health strategies. The timing of efforts to reduce BU acquisition should vary by season. Efforts such as promoting avoidance of biting insects through appropriate clothing or use of insect repellent (*6*) and, potentially, efforts to control mosquito numbers should occur during summer and autumn. Efforts focusing on raising awareness and promoting earlier diagnosis of BU among general practitioners and members of the public should occur during autumn and winter.

Technical Appendix Suburbs or towns considered endemic for Buruli ulcer; region of exposure locations; and severity of Buruli ulcer lesions, Victoria, Australia, 2011–2016.

## References

[1] World Health Organization. Global Health Observatory data [cited 2017 May 22]. http://www.who.int/gho/neglected_diseases/buruli_ulcer/en/

[2] Fyfe JA, Lavender CJ, Handasyde KA, Legione AR, O’Brien CR, Stinear TP, et al. A major role for mammals in the ecology of *Mycobacterium ulcerans.* PLoS Negl Trop Dis. 2010;4:e791. 10.1371/journal.pntd.0000791 20706592 PMC2919402

[3] Wallace JR, Mangas KM, Porter JL, Marcsisin R, Pidot SJ, Howden B, et al. *Mycobacterium ulcerans* low infectious dose and mechanical transmission support insect bites and puncturing injuries in the spread of Buruli ulcer. PLoS Negl Trop Dis. 2017;11:e0005553. 10.1371/journal.pntd.0005553 28410412 PMC5406025

[4] Johnson PD, Azuolas J, Lavender CJ, Wishart E, Stinear TP, Hayman JA, et al. *Mycobacterium ulcerans* in mosquitoes captured during outbreak of Buruli ulcer, southeastern Australia. Emerg Infect Dis. 2007;13:1653–60. 10.3201/eid1311.061369 18217547 PMC3375796

[5] Johnson PD, Lavender CJ. Correlation between Buruli ulcer and vector-borne notifiable diseases, Victoria, Australia. Emerg Infect Dis. 2009;15:614–5. 10.3201/eid1504.081162 19331750 PMC2671447

[6] Quek TY, Athan E, Henry MJ, Pasco JA, Redden-Hoare J, Hughes A, et al. Risk factors for *Mycobacterium ulcerans* infection, southeastern Australia. Emerg Infect Dis. 2007;13:1661–6. 10.3201/eid1311.061206 18217548 PMC3375781

[7] Steffen CM, Smith M, McBride WJ. *Mycobacterium ulcerans* infection in North Queensland: the ‘Daintree ulcer’. ANZ J Surg. 2010;80:732–6. 10.1111/j.1445-2197.2010.05338.x 21040335

[8] Boyd SC, Athan E, Friedman ND, Hughes A, Walton A, Callan P, et al. Epidemiology, clinical features and diagnosis of *Mycobacterium ulcerans* in an Australian population. Med J Aust. 2012;196:341–4. 10.5694/mja12.10087 22432674

[9] Johnson PD, Veitch MG, Leslie DE, Flood PE, Hayman JA. The emergence of *Mycobacterium ulcerans* infection near Melbourne. Med J Aust. 1996;164:76–8. 8569576 10.5694/j.1326-5377.1996.tb101352.x

[10] Francis G, Whitby M, Woods M. *Mycobacterium ulcerans* infection: a rediscovered focus in the Capricorn Coast region of central Queensland. Med J Aust. 2006;185:179–80. 16893367 10.5694/j.1326-5377.2006.tb00516.x

[11] Fyfe JA, Lavender CJ, Johnson PD, Globan M, Sievers A, Azuolas J, et al. Development and application of two multiplex real-time PCR assays for the detection of *Mycobacterium ulcerans* in clinical and environmental samples. Appl Environ Microbiol. 2007;73:4733–40. 10.1128/AEM.02971-06 17526786 PMC1951036

[12] Ross BC, Marino L, Oppedisano F, Edwards R, Robins-Browne RM, Johnson PD. Development of a PCR assay for rapid diagnosis of *Mycobacterium ulcerans* infection. J Clin Microbiol. 1997;35:1696–700. 9196176 10.1128/jcm.35.7.1696-1700.1997 PMC229824

[13] World Health Organization. Laboratory diagnosis of Buruli ulcer: a manual for health care providers: Geneva: The Organization; 2014.

[14] Victorian Department of Health and Human Services. Notification of *Mycobacterium ulcerans* (Buruli ulcer) [cited 2017 Apr 17]. https://www2.health.vic.gov.au/about/publications/formsandtemplates/notification-of-mycobacterium-ulcerans

[15] World Health Organization. Treatment of *Mycobacterium ulcerans* disease (Buruli ulcer): Geneva: The Organization; 2012.

[16] Research Data Australia, Department of the Prime Minister and Cabinet. VIC Suburb/Locality Boundaries—PSMA Administrative Boundaries [cited 2017 May 8]. https://researchdata.ands.org.au/vic-suburblocality-boundaries-administrative-boundaries/644824

[17] Carson C, Lavender CJ, Handasyde KA, O’Brien CR, Hewitt N, Johnson PD, et al. Potential wildlife sentinels for monitoring the endemic spread of human buruli ulcer in South-East australia. PLoS Negl Trop Dis. 2014;8:e2668. 10.1371/journal.pntd.0002668 24498452 PMC3907424

[18] Australian Bureau of Statistics. Population by age and sex, regions of Australia. Canberra (ACT, Australia). Bur. 2013;•••:2014.

[19] Trubiano JA, Lavender CJ, Fyfe JA, Bittmann S, Johnson PD. The incubation period of Buruli ulcer (*Mycobacterium ulcerans* infection). PLoS Negl Trop Dis. 2013;7:e2463. 10.1371/journal.pntd.0002463 24098820 PMC3789762

[20] Loftus MJ, Trubiano JA, Tay EL, Lavender CJ, Globan M, Fyfe JAM, et al. The incubation period of Buruli ulcer (*Mycobacterium ulcerans infection*) in Victoria, Australia - Remains similar despite changing geographic distribution of disease. PLoS Negl Trop Dis. 2018;12:e0006323. 10.1371/journal.pntd.0006323 29554096 PMC5875870

[21] Ablordey A, Swings J, Hubans C, Chemlal K, Locht C, Portaels F, et al. Multilocus variable-number tandem repeat typing of *Mycobacterium ulcerans.* J Clin Microbiol. 2005;43:1546–51. 10.1128/JCM.43.4.1546-1551.2005 15814964 PMC1081385

[22] Australian Bureau of Statistics. Australian demographic statistics. Canberra (ACT, Australia). Bur. 2016;•••:2017.

[23] Edwards JH. The recognition and estimation of cyclic trends. Ann Hum Genet. 1961;25:83–7. 10.1111/j.1469-1809.1961.tb01501.x 13725808

[24] Yerramilli A, Tay EL, Stewardson AJ, Kelley PG, Bishop E, Jenkin GA, et al. The location of Australian Buruli ulcer lesions-Implications for unravelling disease transmission. PLoS Negl Trop Dis. 2017;11:e0005800. 10.1371/journal.pntd.0005800 28821017 PMC5584971

[25] Quek TY, Henry MJ, Pasco JA, O’Brien DP, Johnson PD, Hughes A, et al. *Mycobacterium ulcerans* infection: factors influencing diagnostic delay. Med J Aust. 2007;187:561–3. 18021043 10.5694/j.1326-5377.2007.tb01416.x

[26] Veitch MG, Johnson PD, Flood PE, Leslie DE, Street AC, Hayman JA. A large localized outbreak of *Mycobacterium ulcerans* infection on a temperate southern Australian island. Epidemiol Infect. 1997;119:313–8. 10.1017/S0950268897008273 9440434 PMC2809003

[27] Coutts S, Tay EL. Factors influencing delayed presentation and diagnosis for Buruli ulcer in Victoria, 2011–2016. Presented at: Communicable Diseases Control Conference; 2017 Jun 26–28; Melbourne, Victoria, Australia.

[28] Tai AYC, Athan E, Friedman ND, Hughes A, Walton A, O’Brien DP. Increased severity and spread of *Mycobacterium ulcerans*, southeastern Australia. Emerg Infect Dis. 2018;24:58–64. 10.3201/eid2401.171070 28980523 PMC5749465

[29] Lavender CJ, Fyfe JA, Azuolas J, Brown K, Evans RN, Ray LR, et al. Risk of Buruli ulcer and detection of *Mycobacterium ulcerans* in mosquitoes in southeastern Australia. PLoS Negl Trop Dis. 2011;5:e1305. 10.1371/journal.pntd.0001305 21949891 PMC3176747

[30] O’Brien DP, Wynne JW, Buultjens AH, Michalski WP, Stinear TP, Friedman ND, et al. Exposure risk for infection and lack of human-to-human transmission of *Mycobacterium ulcerans* disease, Australia. Emerg Infect Dis. 2017;23:837–40. 10.3201/eid2305.160809 28418294 PMC5403060

[31] Friedman ND, Athan E, Hughes AJ, Khajehnoori M, McDonald A, Callan P, et al. *Mycobacterium ulcerans* disease: experience with primary oral medical therapy in an Australian cohort. PLoS Negl Trop Dis. 2013;7:e2315. 10.1371/journal.pntd.0002315 23875050 PMC3715400

[32] Pak J, O’Brien DP, Quek T, Athan E. Treatment costs of *Mycobacterium ulcerans* in the antibiotic era. Int Health. 2012;4:123–7. 10.1016/j.inhe.2011.12.005 24029150

