# Supplementary material for: Epidemiology of Buruli Ulcer Infections, Victoria, Australia, 2011–2016
Source: Emerg Infect Dis. 2018 Nov;24(11):1988–97. doi: 10.3201/eid2411.171593 (PMC6199991; doi:10.3201/eid2411.171593)
Supplement: Technical Appendix — Suburbs or towns considered endemic for Buruli ulcer; region of exposure locations; and severity of Buruli ulcer lesions, Victoria, Australia, 2011–2016. [file 17-1593-Techapp-s1.pdf]

Article DOI: https://doi.org/10.3201/eid2411.171593

Epidemiology of Buruli Ulcer Infections in Victoria, Australia, 2011–2016

Technical Appendix

Technical Appendix Table 1. Full list of suburbs or towns considered endemic for Buruli ulcer, classified into regions

Geographic region	Suburbs or towns
Mornington Peninsula	Blairgowrie, Boneo, Cape Shanck, Crib Point, Dromana, Flinders, Hastings, Mornington, Moorooduc, Mount Eliza, Mount Martha, Pearcedale, Portsea, Red Hill, Rosebud, Rye, Safety Beach, Somerville, Sorrento, St Andrews, Tootgarook
Bellarine Peninsula	Barwon Heads, Bellarine, Clifton Springs, Drysdale, Leopold, Ocean Grove, Point Lonsdale, Portarlington, Queenscliff, St Leonards
Frankston area	Carrum, Frankston, Langwarrin, Patterson Lakes, Seaford
South-eastern bayside suburbs	Beaumaris, Black Rock, Brighton, Chelsea, Cheltenham, Hampton, Highett, Mentone, Parkdale, Sandringham

Technical Appendix Table 2. Region of exposure locations by year (all categories of certainty)*

Year	Mornington Peninsula	Bellarine Peninsula	Frankston	Gippsland	Philip Island	South-eastern bayside suburbs	Interstate	Total
2011	6	61	8	0	1	3	1	80
2012	8	59	7	0	3	4	0	81
2013	21	36	7	1	0	4	0	69
2014	37	41	10	2	0	6	0	96
2015	64	27	13	1	0	7	0	112
2016	137	25	16	0	0	13	1	192
TOTAL	273	249	61	4	4	37	2	630

*The number of exposures is greater than the number of cases due to those who traveled to multiple regions.

Technical Appendix Table 3. Severity of lesions for Buruli ulcer cases, Victoria, by year

Year	Total	WHO Category I	WHO Category II	WHO Category III	Not stated	WHO Category II & III n (%)*
2011	80	51	10	7	12	17/68 (25.0)
2012	77	55	7	4	11	11/66 (16.7)
2013	65	43	9	5	8	14/57 (24.6)
2014	89	63	12	9	5	21/84 (25.0)
2015	107	70	12	8	17	20/90 (22.2)
2016	182	133	21	4	24	25/158 (15.8)

*Denominator excludes cases with missing data on severity of lesion classified according to WHO Category. Chi-squared test for trend p-value = 0.2319, comparing the changes in proportion of cases with severe disease (WHO Category II and III) over time.
